# The Societal Burden of Breast Cancer in Working-Age Women in Croatia: A Multicentre Cross-Sectional Study

**DOI:** 10.3390/healthcare14121693

**Published:** 2026-06-12

**Authors:** Vid Duplančić, Ana Bobinac, Luka Vončina, Katarina Hraste, Ana Tečić Vuger, Robert Šeparović, Eduard Vrdoljak

**Affiliations:** 1 Department of Radiotherapy and Oncology, University Hospital Center Split, School of Medicine, University of Split, 21000 Split, Croatia; 2 Faculty of Economics and Business, University of Rijeka, 51000 Rijeka, Croatia; 3 Faculty of Health Studies, University of Rijeka, 51000 Rijeka, Croatia; 4 Division for Medical Oncology, University Hospital for Tumors, Sestre Milosrdnice University Hospital Center, 10000 Zagreb, Croatia

**Keywords:** breast neoplasms, cost of illness, quality of life, absenteeism, presenteeism, caregivers, cross-sectional studies, Croatia, technology assessment, biomedical

## Abstract

**Highlights:**

**What are the main findings?**

- Breast cancer in working-age women in Croatia carries a burden that extends beyond medical care.
- Economic indirect costs were estimated at €8899 per patient-year (95% CI €7835–€9963) and consisted of productivity loss and informal caregiving. Monetised HRQoL/welfare loss was reported separately at €2550 per patient-year (95% CI €2083–€3017).

**What are the implications of the main findings?**

- Public health planning, survivorship care and resource allocation should take these wider costs into account.
- Omitting indirect and welfare losses may underestimate the value of oncology interventions.

**Abstract:**

Background/Objectives: Breast cancer affects working-age women not only through treatment and survival but also through health-related quality of life (HRQoL), work capacity and informal caregiving needs. Evidence from Central and Eastern Europe remains limited. This study estimated the indirect societal burden of breast cancer among working-age women in Croatia and reported economic indirect costs separately from monetised HRQoL/welfare loss. Methods: A multicentre cross-sectional study conducted in 2024 included women aged 18–65 years receiving outpatient oncology care at two tertiary centres in Croatia. HRQoL was assessed with the EuroQol five-dimension five-level instrument (EQ-5D-5L) and compared with Croatian general-population norms. Utility decrements were annualised and monetised using a national willingness-to-pay threshold of €17,000 per quality-adjusted life year (QALY). Work productivity impairment was measured using the Work Productivity and Activity Impairment: General Health (WPAI:GH) questionnaire and valued, together with informal care, using the human-capital approach. Deterministic sensitivity analyses and approximate 95% confidence intervals were used to show how the estimates changed under key assumptions. Results: A total of 271 women participated (mean age 51.3 years among age-eligible records). Mean EQ-5D-5L utility was 0.76 versus 0.91 in the general population, corresponding to an annual QALY loss of 0.15 and a monetised HRQoL/welfare loss of €2550 per patient-year (95% CI €2083–€3017). Among employed participants, mean overall work productivity loss was 43.9% (842.9 h/year), equivalent to €7333 annually (95% CI €6311–€8355). Informal caregiving was reported by 54.7% of participants, with mean annual costs of €1566 (95% CI €1269–€1863). Economic indirect costs were €8899 per patient-year (95% CI €7835–€9963). In an extended welfare-inclusive scenario, the estimated burden was €11,449 per patient-year (95% CI €10,287–€12,611), corresponding to an illustrative national estimate of €86 million (95% CI €77–€95 million; 0.11% of gross domestic product). Conclusions: Breast cancer in working-age women imposes a substantial societal burden in Croatia, driven by reduced HRQoL, productivity losses and informal caregiving needs. These findings support taking societal burden into account in public health planning, survivorship care and health policy decision-making.

## 1. Introduction

Breast cancer is the most common malignancy among women worldwide and a leading cause of premature mortality and reduced health-related quality of life (HRQoL) [1,2]. The World Health Organisation (WHO) estimated approximately 670,000 breast cancer deaths globally in 2022 [2]. In Croatia, GLOBOCAN 2022 estimated 3108 new breast cancer cases and 12,276 women living with a breast cancer diagnosis within 5 years, while recent European cancer surveillance data identified breast cancer as the leading incident cancer among Croatian women [3,4,5,6]. Beyond its clinical consequences, breast cancer imposes substantial psychological, social, and economic burdens on patients, survivors, and their families [7,8,9,10,11].

Because detection and treatment have improved, the consequences of breast cancer increasingly extend beyond acute treatment and mortality. Women living with and beyond breast cancer may experience persistent physical, psychological, social, and functional sequelae during treatment and long after treatment completion, and unmet supportive care needs remain common across the survivorship trajectory [10,11,12,13]. These issues are especially relevant for women of working age, for whom return to work and preservation of work ability are central components of recovery, social participation, and household economic stability. Systematic reviews have identified multiple barriers to return to work among breast cancer survivors and European cancer survivors more broadly, including treatment intensity, fatigue, impaired functioning, lower education, and job-related factor [11,14]. Among working-age women, these burdens extend to reduced labour-market participation, productivity losses due to absenteeism and presenteeism, and caregiving demands within households [15,16,17]. As a substantial proportion of breast cancer cases occurs during prime working years in European and Croatian data [4,6], the consequences are not limited to healthcare use but also affect family welfare and economic productivity.

From a public health perspective, the burden of breast cancer extends beyond healthcare systems to workforce participation, household welfare, and demand for survivorship support services. These issues are especially relevant in ageing populations and resource-constrained systems, including parts of Central and Eastern Europe (CEE), where patient-reported outcomes and broader societal burden estimates remain underrepresented [13,15,18]. Because health systems, labour markets, and social support structures differ across Europe, findings from Western European settings may not be directly transferable to Croatia or other CEE countries [5,18].

Accordingly, the burden of breast cancer should not be viewed only through direct medical expenditure. A broader societal perspective is relevant because illness can affect not only healthcare utilisation but also HRQoL, productivity, and the need for unpaid informal care. The Second Panel on Cost-Effectiveness in Health and Medicine recommends that broader societal consequences be considered alongside healthcare-sector consequences in economic analyses [19], while cancer caregiving literature consistently shows that informal caregivers provide substantial practical and economic support while also experiencing meaningful burden [17,20]. Understanding indirect costs is therefore important for health economic evaluation and policy prioritisation, because analyses limited to direct medical expenditure may underestimate the full welfare impact of cancer [15,19].

At the same time, the available evidence remains uneven across Europe. Population-based studies have shown that cancer generates substantial economic burden across the European Union (EU) [15], and recent analyses from CEE have highlighted the productivity losses associated with premature breast cancer mortality [21]. However, the identifiable regional evidence base is still weighted toward mortality-based or aggregate estimates, whereas patient-level studies that jointly examine HRQoL loss, work impairment, and informal caregiving remain comparatively limited in the Croatian and wider CEE context. Local estimates are therefore needed because the composition and magnitude of indirect burden are shaped by country-specific patterns of survivorship, employment, wage levels, and family caregiving [5,18].

To our knowledge, no Croatian societal cost-of-illness analysis of breast cancer among working-age women has jointly estimated productivity loss, informal caregiving costs, and monetised health utility loss. Unlike many burden studies that focus primarily on a single non-medical component, this analysis combines all three within a single societal framework. Against this background, the present study aimed to estimate the indirect societal burden of breast cancer among working-age women in Croatia by combining three patient-reported components: HRQoL decrement, work productivity impairment, and informal caregiving. By focusing on women treated in routine oncology practice, the study seeks to contribute evidence that is relevant to survivorship care, public health planning, and broader value assessment beyond direct healthcare costs [5,19,21]. We hypothesised that breast cancer among working-age women in Croatia would be associated with substantial indirect societal costs driven primarily by productivity loss and reduced HRQoL.

## 2. Materials and Methods

This multicentre cross-sectional study was conducted in 2024 at two tertiary oncology centres in Croatia: University Hospital Centre Split and University Hospital for Tumours, Sestre Milosrdnice University Hospital Centre, Zagreb.

Eligible participants were women aged 18–65 years with a confirmed diagnosis of breast cancer who were managed in an outpatient ambulatory oncology setting at the participating centres, either (i) while receiving ongoing systemic anticancer treatment (including endocrine therapy) or (ii) during follow-up after completing active systemic anticancer treatment within the preceding 24 months. The 24-month criterion applied to women not receiving ongoing systemic treatment at the time of recruitment (time since diagnosis refers to the time from first breast cancer diagnosis to questionnaire completion, whereas the 24-month criterion refers to completion of the most recent active systemic anticancer treatment episode). Eligibility was not restricted by disease stage or tumour immunophenotype. Exclusion criteria included age <18 years, lack of written informed consent and severe comorbidities or psychiatric conditions that could compromise the validity of self-reported data. As noted previously, patients whose most recent anticancer treatment had been completed more than 24 months before recruitment were excluded. No formal a priori sample-size calculation was performed because the study was designed as an exploratory burden-of-illness analysis.

### 2.1. Data Collection

Potential participants were identified through hospital records at both study sites and contacted by telephone if eligible. A consecutive convenience sampling approach was used, whereby eligible patients attending outpatient oncology visits during the study period were invited to participate. Women who agreed to participate completed a digital questionnaire during their next outpatient visit. At that visit, written informed consent was obtained for both questionnaire participation and extraction of relevant clinical data from hospital records. Participants completed the questionnaire independently (to avoid introducing additional bias) and were informed that their responses would not affect their clinical care. The number of screened, contacted and declining patients was not systematically recorded; therefore, participants could not be formally compared with non-participants and this is acknowledged as a limitation.

Each participant was assigned a study-specific identification code. Clinical data, including disease stage and most recent treatment, were retrieved by authorised personnel and entered into a secure database. Patient-reported data were collected through an electronic questionnaire without personally identifying information. Clinical and survey data were linked using unique identification codes, with access restricted to authorised study staff. Data were stored on password-protected systems and basic data quality checks, including range and completeness checks, were performed at data entry.

### 2.2. Health-Related Quality of Life Assessment and Monetary Valuation

Health-related quality of life (HRQoL) was measured using the EuroQol five-dimension five-level instrument (EQ-5D-5L) [22]. Because no Croatian EQ-5D-5L value set is available, Croatian responses were converted into utility scores using the Slovenian value set developed by Prevolnik Rupel and Ogorevc [23]. This value set was selected because it is a directly elicited EQ-5D-5L value set from a neighbouring Central/Eastern European country. Utility values were used to estimate welfare loss relative to EQ-5D-5L scores from a representative sample of Croatian women of the same age, collected in 2022 and described elsewhere [24]. These general-population data were not collected as part of the present breast cancer study but served as an external normative comparator.

Given the cross-sectional design, the utility decrement (general-population mean minus patient mean) was annualised by assuming that the gap persisted over one year. The monetised utility loss should therefore be read as an approximate scenario-based one-year welfare loss rather than a longitudinal estimate; it was reported separately from productivity and informal-care costs because these concepts may overlap. Monetary valuation was calculated by multiplying the utility decrement by a Croatian willingness-to-pay (WTP) threshold of €17,000 per quality-adjusted life year (QALY), reported in the literature [25].

### 2.3. Productivity Measurement

Productivity was assessed using the Work Productivity and Activity Impairment: General Health (WPAI:GH) questionnaire [26]. The instrument captures impairment over the previous seven days across four domains: absenteeism (% work time missed), presenteeism (% impairment while working), overall work productivity loss (combined absenteeism and presenteeism) and activity impairment (% limitation in daily activities). In the base case, observed weekly impairment was annualised by assuming that the 7-day recall period reflected typical work impairment. Standard WPAI:GH scoring methods were applied [27]. All indices range from 0 to 100%, with higher values indicating greater impairment.

Productivity loss was monetised using the human-capital approach, valuing lost work time as foregone earnings [28]. Annual lost hours were calculated by applying WPAI:GH percentages to total annual working time (1920 h) and valued using the average net wage for Croatian women (approximately €1392 per month; €8.70 per hour) [29].

The WPAI:GH questionnaire was translated from English to Croatian using a forward–backward procedure by two independent bilingual translators, with reconciliation by the research team. The general-health version was selected because it is an internationally recognised instrument for measuring absenteeism, presenteeism, overall work productivity loss and activity impairment across health conditions [26,27].

### 2.4. Informal Care

Informal care was assessed using two items on receipt and frequency of care from family members or friends over the past 12 months. Frequency was recorded as an ordinal variable (1 = never to 7 = daily) and converted into approximate monthly hours assuming an average duration of 2 h per care episode (e.g., less than once a month = 0.5 h/month; daily = 60 h/month). Because this conversion required assumptions about episode duration and category-to-hours mapping, informal-care estimates were treated as approximate and the assumed duration per episode was varied in sensitivity analysis.

Informal care was valued using the same human-capital approach as productivity losses [28,29]. Because caregiver characteristics were not collected, care time was valued using the same sex-specific wage input applied in productivity costing (female net hourly earnings), keeping valuation consistent across indirect cost components.

### 2.5. Statistical Analysis

Descriptive statistics (mean, standard deviation [SD], range and, where appropriate, median and interquartile range [IQR]) were calculated for HRQoL outcomes. Age was stratified into quintiles based on its empirical distribution to create five groups of approximately equal size. Between-group differences were assessed using the Mann–Whitney U test for two groups and the Kruskal–Wallis test for multiple groups. All statistical analyses were performed using Stata version 19 (StataCorp LLC, College Station, TX, USA). A two-sided *p*-value < 0.05 was considered statistically significant.

Results were expressed as mean annual cost per patient and extrapolated to the national level by multiplying per-patient values by the estimated number of working-age women living with breast cancer. This population was approximated using GLOBOCAN 2022 5-year prevalence data for Croatia (12,276 cases, all ages) and an assumed working-age share of approximately 61%, yielding ~7500 women [3].

The analyses used available cases for each outcome; because of missing values or item non-response, denominators vary across analyses (Supplementary Table S1). Approximate 95% confidence intervals were calculated as mean ± 1.96 standard errors using the available-case mean, SD and denominator for each component. For monetised HRQoL/welfare loss, the standard error of the utility decrement was calculated from the patient and general-population samples; for productivity loss and informal care, standard errors were based on the corresponding available-case cost distributions. For economic indirect costs and the extended welfare-inclusive scenario, component variances were combined assuming independence. These intervals should therefore be interpreted as approximate uncertainty intervals around mean burden estimates (Supplementary Table S2).

Deterministic one-way sensitivity analysis varied one parameter at a time while holding all other base-case inputs constant to explore the impact of different determinants on the extended welfare-inclusive burden estimate. The base-case assumptions were: WTP threshold €17,000/QALY, hourly wage €8.70, annual working time 1920 h, full-year productivity persistence of the WPAI-derived weekly impairment, informal-care duration of 2 h per episode and working-age share of 61% for national extrapolation. Low and high scenarios were prespecified as symmetric ±25% variations around the base-case inputs (Table 6).

Exploratory subgroup analyses compared outcomes by age group and metastatic versus non-metastatic disease in the main tables; additional subgroup analyses are presented in Supplementary Table S3 (active/maintenance treatment versus completed treatment/follow-up and time since diagnosis [<2, 2–5 and >5 years], using the same non-parametric testing approach as the main unadjusted analyses). These subgroup analyses were interpreted cautiously because of small subgroup sizes. We also ran exploratory covariate-adjusted analyses as supportive internal-validity checks; they were not used to derive the main burden estimates. Linear models with robust standard errors were used for continuous HRQoL, productivity and cost outcomes and logistic regression was used for informal-care receipt. Models adjusted for age, metastatic disease, active/maintenance treatment, time since diagnosis, higher education and study centre and are reported in Supplementary Table S4. Completer versus non-completer comparisons were performed for the EQ-5D/VAS, WPAI and informal-care modules using available clinical and sociodemographic characteristics to assess potential module-level non-response bias; these analyses are reported in Supplementary Table S5.

The study was conducted in accordance with the Declaration of Helsinki and approved by the relevant institutional ethics committees.

## 3. Results

### 3.1. Participant Characteristics

A total of 271 women participated in the study (Table 1). The mean age was 51.35 years (SD 8.52) and the mean time since diagnosis was 4.5 years (SD 3.57). Overall, 19.2% of participants had metastatic disease.

Most participants had secondary or higher education and 70.8% were employed (including those on sick leave). A negative impact of breast cancer on household finances was reported by 45.3% of participants, while 21.2% indicated that it was fairly or extremely difficult to make ends meet.

Questionnaire module completion varied so not all participants contributed data to every outcome. Sample sizes therefore differ across analyses and are shown in the relevant tables; participant-flow and module-specific denominators are summarised in Supplementary Table S1. Completer/non-completer comparisons are reported in Supplementary Table S5; the main observed differences were that EQ-5D/informal-care module completers were more often in active/maintenance treatment than non-completers (85.4% vs. 63.6%) and WPAI complete cases were more often highly educated than WPAI non-completers (58.5% vs. 27.8%). Because screened, contacted and declined counts were not available, non-participant selection bias could not be quantified.

### 3.2. Health-Related Quality of Life

**Figure 1:**
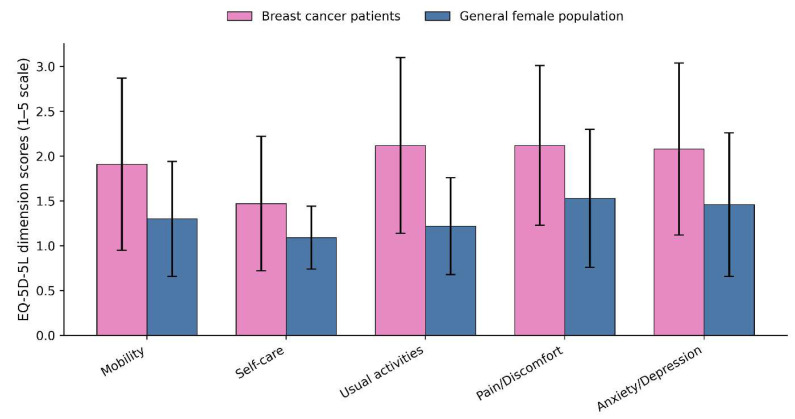
EuroQol five-dimension five-level instrument (EQ-5D-5L) dimension scores in breast cancer patients (*n* = 233) and the general female population aged 18–65 years. Note: Error bars on Figure 1 represent standard deviations.

Health-related quality-of-life outcomes are presented in Table 2. Mean EQ-5D-5L utility was 0.76 in breast cancer patients and 0.91 in the Croatian female general population, corresponding to a utility decrement of 0.15. Mean EQ-VAS scores were 66.17 and 83.99, respectively. Scores also differed significantly across all five EQ-5D-5L dimensions (*p* < 0.01 for all comparisons) (Figure 1).

For economic valuation, the observed cross-sectional utility gap of 0.15 was treated as an annualised QALY loss of 0.15 per patient-year, assuming that the decrement persisted over one year. Applying the base-case WTP threshold (€17,000 per QALY), this corresponds to a monetised HRQoL/welfare loss of €2550 per patient-year (approximate 95% CI €2083–€3017).

Age-stratified analyses showed a decline in HRQoL with increasing age among breast cancer patients (Table 2). Mean EQ-5D-5L index scores decreased from 0.82 in the youngest age group (24–43 years) to 0.70 in the oldest group (60–65 years) (Kruskal–Wallis *p* = 0.03). EQ-VAS scores showed a similar pattern, although the trend was not statistically significant (*p* = 0.08). In all age groups, patient utility values remained lower than those of the general population.

As shown in Table 3, metastatic disease was associated with lower HRQoL. Mean EQ-5D-5L index and EQ-VAS scores were 0.70 and 58.23 in metastatic patients, compared with 0.77 and 67.96 in non-metastatic patients (*p* = 0.03 and *p* = 0.01, respectively).

### 3.3. Productivity

**Figure 2:**
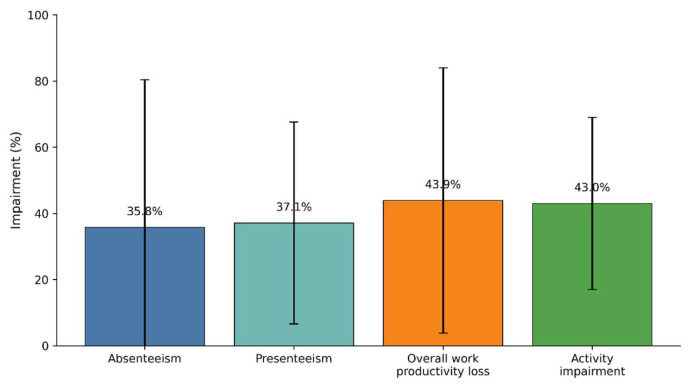
Work Productivity and Activity Impairment: General Health (WPAI:GH) outcomes among employed breast cancer patients. Note: Error bars represent standard deviations.

Table 4 summarises WPAI:GH outcomes. Mean overall work productivity loss was 43.9% (SD 40.1), corresponding to 842.9 h (105 workdays) lost annually per employed patient and €7333 per patient per year (approximate 95% CI €6311–€8355) (Figure 2).

Mean absenteeism was 35.8% (SD 44.6), mean presenteeism was 37.1% (SD 30.5) and mean activity impairment was 43.0% (SD 26.0).

Considered separately, absenteeism corresponded to 687 annual hours (€5980) and presenteeism to 457 h (€3979); however, total costs were estimated using overall productivity loss to avoid double-counting.

No statistically significant differences were observed between metastatic and non-metastatic patients across WPAI:GH domains (Table 4; *p* = 0.11–0.89) or across age groups (Table 4; *p* = 0.54–0.89).

### 3.4. Informal Care

Overall, 54.7% of respondents reported receiving informal care from family or friends during the past year (Table 5). The mean amount of care was equivalent to 22.5 working days annually (SD 33.4), corresponding to €1566 per patient per year using the human-capital approach (€8.70/h; approximate 95% CI €1269–€1863).

Informal care was more frequent among patients with metastatic disease (65.9% vs. 52.1%) and associated with a higher number of care days (27.8 vs. 21.3 days/year), although these differences were not statistically significant (*p* = 0.10 and *p* = 0.23, respectively). No significant differences were observed across age groups for either outcome (*p* > 0.60; Table 5).

When monetised HRQoL/welfare loss was kept separate from resource and opportunity costs, economic indirect costs amounted to €8899 per patient-year (productivity loss plus informal care; approximate 95% CI €7835–€9963; Supplementary Table S2). Adding all components in an extended welfare-inclusive scenario yielded €11,449 per patient-year (approximate 95% CI €10,287–€12,611; Supplementary Table S2). The deterministic one-way sensitivity analysis is presented in Table 6.

### 3.5. Sensitivity Analysis

Table 6 summarises the deterministic one-way sensitivity analysis around the extended welfare-inclusive burden estimate. The low and high scenarios correspond to symmetric ±25% variations around each base-case input (extended welfare-inclusive burden estimate €11,449 per patient-year). The largest per-patient impact was observed for the hourly wage input, followed by the productivity-related inputs (WPAI annualisation/productivity persistence and annual working hours), the WTP threshold and informal-care duration. Exploratory covariate-adjusted models presented in Supplementary Table S4 suggested lower utility among older patients and those in active/maintenance treatment and lower WPAI activity impairment among women with higher education and longer time since diagnosis. These patterns were consistent with expected clinical and socioeconomic gradients and were used only as checks of internal consistency, not as inputs into the burden estimates.

## 4. Discussion

This study shows a substantial indirect burden of breast cancer among working-age women in Croatia, reflected in reduced health-related quality of life (HRQoL), impaired work capacity and reliance on informal caregiving. The impairment observed in pain/discomfort and anxiety/depression is consistent with survivorship literature documenting ongoing physical and psychological consequences after treatment [8,12,13]. These findings suggest that functional and emotional outcomes remain clinically relevant beyond active therapy.

Croatia’s pattern of survivorship burden appears broadly similar to that reported in Western European cohorts but the monetised magnitude is shaped by structural differences between CEE and Western Europe. In Dutch breast cancer patients, mean EQ-5D-5L indices around 0.80–0.85 during the first year after surgery have been observed [30]. At the same time, Western European general-population EQ-5D-5L norms can be high; for example, a Dutch female normative mean utility of 0.911 has been reported [31], meaning that even moderate patient decrements can translate into meaningful welfare losses. Cross-country comparisons should still be interpreted cautiously because EQ-5D utilities depend on country-specific value sets and empirical work shows that tariffs are correlated but not interchangeable [32].

The findings also point to the economic and public-health dimensions of survivorship. Valuing productivity losses with the human-capital approach [28], together with nationally representative wage data [29], places work impairment in a broader economic context. Adding monetised QALY losses extends the perspective beyond labour-market effects and captures welfare losses that are often left out of traditional cost-of-illness studies [33]. This is consistent with European evidence showing that non-health-care costs, including informal care and productivity loss, can be substantial and sometimes comparable in magnitude to direct cancer-care spending [34]. From a public health perspective, the observed levels of work impairment and caregiving need point to sustained functional limitations that may affect workforce participation, household welfare and service needs in ageing and resource-constrained settings [15,34].

At the same time, monetised HRQoL loss, productivity loss and informal caregiving should not be treated as automatically additive. EQ-5D-5L captures usual activities, pain/discomfort and anxiety/depression, which may overlap conceptually with work impairment and dependency on informal care. For this reason, the revised analysis treats productivity plus informal care as the primary economic indirect-cost estimate and presents the sum including monetised HRQoL loss only as an extended welfare-inclusive scenario.

Work-related outcomes are particularly sensitive to national labour-market conditions and costing methods. In France, a prospective multicentre cohort of early-stage breast cancer patients found that 93% took sick leave, with a mean of 186 days in the first year after diagnosis and that indirect costs differed markedly according to valuation method (human capital €22,722 vs. friction cost €7724 per patient) [35]. In our sample, the employment rate was broadly comparable with national female labour-market statistics, whereas the observed sick-leave rate was substantially higher than the national temporary work incapacity rate [36,37]. These comparisons should be interpreted cautiously but they illustrate that indirect-cost estimates are influenced both by disease burden and by the methodological choice used to value lost productivity [28,35].

The high sick-leave rate is important methodologically because WPAI:GH has a 7-day recall window. The recruitment week may therefore have captured a period of elevated impairment and should not be assumed to represent the entire work year without qualification. Aggregate Croatian administrative sick-leave data are not diagnosis-, sex- and age-matched to this cohort, so they were not used for formal calibration; instead, shorter productivity-persistence scenarios were included in sensitivity analysis.

The results also support integrating structured psychosocial support and routine mental health screening into survivorship care pathways. This is consistent with American Society of Clinical Oncology guidance, which recommends structured screening and evidence-based psychosocial interventions to improve depression and anxiety outcomes in adult cancer survivors [38]. Systematic HRQoL monitoring may help identify patients at risk of persistent distress earlier and support targeted interventions that improve long-term outcomes.

The informal-care findings are also relevant internationally, particularly in CEE settings where families may carry a larger share of care when formal services are constrained. European evidence indicates wide cross-country variation in informal caregiving prevalence and intensity and suggests that stronger family-care norms and lower formal long-term-care provision are associated with more intensive caregiving [39]. European analyses also show that informal caregivers for cancer patients experience their own HRQoL and productivity impairments, implying that informal care can generate secondary welfare losses beyond the caregiver’s time input [40]. These findings support including informal care in societal burden analyses and in survivorship service planning.

Several methodological limitations should be considered. The cross-sectional design precludes causal inference and does not allow assessment of changes over the disease trajectory [41]. Annualised productivity costs and monetised utility losses were derived from a 7-day WPAI:GH recall window and cross-sectional EQ-5D-5L values; they therefore represent approximate one-year burden estimates rather than longitudinal trajectories. The high proportion of employed participants reporting sick leave during the recruitment week reinforces the possibility that the observed week was not representative of the entire year. The WPAI:GH instrument captures impairment attributable to general health rather than breast cancer specifically. Although severe comorbidities were excluded, residual attribution to other health problems cannot be ruled out and the Croatian translation was not formally cognitively validated in breast cancer patients. Recruitment from two tertiary oncology centres may limit generalisability and may overrepresent patients with more complex treatment pathways, while the number of screened, contacted and declining patients was not systematically recorded. Module-specific non-response was also not fully random: EQ-5D/informal-care module completers were more often in active/maintenance treatment, while WPAI complete cases were more often highly educated (Supplementary Table S5). This may have influenced available-case estimates and supports cautious interpretation of the results. Productivity loss and informal care were self-reported, which introduces the possibility of recall and reporting bias; prior work has shown discrepancies between self-reported and employer-recorded absence data [42]. Informal care time was estimated from frequency categories using assumed duration values, an approach consistent with established valuation methods but still approximate [43,44]. The absence of a Croatian EQ-5D-5L value set is an additional limitation, because utility estimates may vary when different national tariffs are applied [34]. No formal a priori sample-size calculation was performed because the study was designed as an exploratory burden-of-illness analysis. Finally, monetised HRQoL/welfare loss may overlap conceptually with productivity and informal-care costs; therefore, the extended total should be interpreted cautiously and the economic indirect-cost estimate should be considered the primary resource/opportunity-cost result.

Although not directly assessed in this study, treatment patterns and toxicity profiles are likely to influence the long-term indirect burden of breast cancer. More intensive therapy and residual disease may prolong work absence and worsen survivorship outcomes [45,46,47,48,49]. Future studies should examine how treatment pathway, recurrence risk and treatment-related toxicity shape productivity loss, HRQoL and informal-care needs over time.

From a CEE-versus-Western Europe perspective, access patterns and time to coverage for high-benefit cancer medicines are likely to influence long-term survivorship and functional outcomes [50]. Comparative analyses by the Organisation for Economic Co-operation and Development indicate substantial cross-country variation in health-system performance, access to innovative therapies and healthcare resource allocation across Europe [51]. In this context, including indirect costs and welfare losses in health technology assessment (HTA) may be particularly relevant for CEE decision-making, because interventions that reduce recurrence, treatment toxicity or persistent symptoms may generate societal value through improved productivity and reduced caregiver burden—effects that may be insufficiently captured under narrower payer perspectives [52]. This supports a broader societal perspective in policy appraisal and survivorship planning.

## 5. Conclusions

Breast cancer among working-age women in Croatia imposes a substantial societal burden beyond direct medical expenditure. Economic indirect costs were driven mainly by productivity loss and informal caregiving and were estimated at €8899 per patient-year (95% CI €7835–€9963). Monetised HRQoL/welfare loss represented an additional component of €2550 per patient-year (95% CI €2083–€3017) and was reported separately to reduce the risk of double counting.

In an extended welfare-inclusive scenario, the combined burden was €11,449 per patient-year (95% CI €10,287–€12,611), corresponding to an illustrative national estimate of approximately €86 million (95% CI €77–€95 million). Because these estimates are annualised from cross-sectional data and depend on valuation and persistence assumptions, they should be interpreted as scenario-based estimates rather than precise observed annual costs.

These findings show that the consequences of breast cancer extend beyond healthcare budgets to labour markets, households and broader welfare. Future longitudinal studies with administrative linkage and disease-stage-specific follow-up are needed to quantify how productivity loss, HRQoL and informal care vary across treatment pathways and survivorship trajectories.

## Figures and Tables

**Table 1:** Sociodemographic and economic characteristics of the study sample (*N* = 271).

Variable	Category	*n* (%)/Mean (SD)
Age (years)	Mean (SD)	51.35 (8.52)
Education level	Primary or less	9 (3.8%)
	Secondary school	121 (51.3%)
	Undergraduate degree	87 (36.9%)
	Postgraduate degree	19 (8.0%)
Employment status	Employed (incl. sick leave)	165 (70.8%)
	– Of which reporting being on sick leave in the previous week (sick leave rate)	92 (56% of 165)
	– Mean duration of ongoing sick leave among those on sick leave (days)	14.3
	– Persons being on sick leave the entire previous week	45 (49% of 92)
	Retired	29 (12.5%)
	Unemployed	15 (6.4%)
	Housework	19 (8.1%)
	Permanently unfit for work	5 (2.1%)
Negative impact on marital status	Yes	20 (8.5%)
	No	216 (91.5%)
Negative impact on household finances	Yes	107 (45.3%)
	No	129 (54.7%)
Household ability to make ends meet	Extremely easy	3 (1.3%)
	Fairly easy	32 (13.6%)
	Neither easy nor difficult	140 (59.3%)
	Fairly difficult	46 (19.5%)
	Extremely difficult	4 (1.7%)
	Cannot decide	11 (4.7%)
Time since diagnosis (years)	Mean (SD)	4.5 (3.57)
Metastatic disease	No	219 (80.8%)
	Yes	52 (19.2%)

Note: Percentages are calculated among respondents with available data for each variable; denominators therefore vary because of item non-response. SD, standard deviation.

**Table 2:** Health-related quality of life in breast cancer patients and external Croatian female general-population normative values, overall and by age group.

Variable/Group	General P471.	Breast Cancer Patients Mean (SD)	Test Statistic	*p*-Value
Overall (*n* = 471 vs. *N* = 233)				
EQ-5D-5L index	0.91 (0.14)	0.76 (0.19)		<0.01
EQ-VAS	83.99 (16.42)	66.17 (23.09)		<0.01
Mobility	1.30 (0.64)	1.91 (0.96)		<0.01
Self-care	1.09 (0.35)	1.47 (0.75)		<0.01
Usual activities	1.22 (0.54)	2.12 (0.98)		<0.01
Pain/Discomfort	1.53 (0.77)	2.12 (0.89)		<0.01
Anxiety/Depression	1.46 (0.80)	2.08 (0.96)		<0.01
Age-stratified EQ-5D index			KW χ^2^ = 10.38	0.03
24–43 (*n* = 248 vs. *N* = 35)	0.95 (0.10)	0.82 (0.15)		
44–48 (*n* = 71 vs. *N* = 53)	0.91 (0.11)	0.79 (0.15)		
49–54 (*n* = 45 vs. *N* = 56)	0.86 (0.16)	0.78 (0.17)		
55–59 (*n* = 56 vs. *N* = 41)	0.84 (0.16)	0.71 (0.22)		
60–65 (*n* = 51 vs. *N* = 48)	0.82 (0.21)	0.70 (0.23)		
Age-stratified EQ-VAS			KW χ^2^ = 8.35	0.08
24–43 (*n* = 248 vs. *N* = 35)	89.06 (13.61)	72.00 (25.08)		
44–48 (*n* = 71 vs. *N* = 53)	83.61 (16.51)	65.51 (24.40)		
49–54 (*n* = 45 vs. *N* = 56)	78.62 (16.88)	69.91 (19.41)		
55–59 (*n* = 56 vs. *N* = 41)	76.73 (15.81)	61.17 (25.50)		
60–65 (*n* = 51 vs. *N* = 48)	72.55 (19.11)	62.54 (21.10)		

Note: Values are mean (SD). General-population values were obtained from a previously published representative Croatian population survey restricted to women aged 18–65 years and were used as an external normative benchmark. These participants were not recruited as controls in the present study; therefore, the comparison should not be interpreted as a matched case–control comparison or as evidence of causality. Between-group comparisons were assessed using the Mann–Whitney U test, and age-group differences using the Kruskal–Wallis test (KW). EQ-5D-5L utilities in both datasets were derived using the same Slovenian EQ-5D-5L value set. EQ-VAS, EuroQol Visual Analogue Scale; SD, standard deviation.

**Table 3:** Health-related quality of life by disease stage.

Variable	Non-Metastatic (*n* = 190) Mean (SD)	Metastatic (*n* = 43) Mean (SD)	KW χ^2^ (df = 1)	*p*-Value
EQ-5D-5L index	0.77 (0.18)	0.70 (0.21)	4.93	0.03
EQ-VAS	67.96 (22.52)	58.23 (24.16)	6.69	0.01

Note: Values are mean (SD). Between-group differences were analysed using the Kruskal–Wallis test. EQ-VAS, EuroQol Visual Analogue Scale; SD, standard deviation.

**Table 4:** Work Productivity and Activity Impairment: General Health (WPAI:GH) outcomes among employed breast cancer patients.

WPAI Domain	Questionnaire Item (WPAI:GH)	Group	*n*	Mean (SD)	Median (IQR)	Statistic	*p*-Value
Absenteeism (% work time missed)	Q1. “During the past 7 days, how many hours did you miss from work because of health problems?”	All employed	152	35.8 (44.6)	5 (100)		
		Non-metastatic	136	33.7 (44.0)	5 (100)	Mann–Whitney *z* = –1.61	0.11
		Metastatic	16	53.3 (47.0)	12.5 (100)		
Presenteeism (% impairment while working)	Q2. “During the past 7 days, how much did your health problems affect your productivity while you were working?” (0 = no effect; 10 = completely prevented working)	All employed	156	37.1 (30.5)	40 (50)		
		Non-metastatic	140	36.5 (29.9)	40 (50)	Mann–Whitney *z* = –0.66	0.51
		Metastatic	16	42.5 (35.7)	40 (65)		
Overall work productivity loss (%)	Calculated from Q1 and Q2: 1 − [(1 − absenteeism/100) × (1 − presenteeism/100)] × 100	All employed	165	43.9 (40.1)	40 (100)		
		Non-metastatic	146	43.6 (40.3)	40 (100)	Mann–Whitney *z* = –0.13	0.89
		Metastatic	19	45.7 (41.1)	40 (100)		
Activity impairment (% limitation in daily activities)	“During the past 7 days, how much did your health problems affect your ability to do your regular daily activities, other than work at a job?” (0 = no effect; 10 = completely prevented)	All employed	165	43.0 (26.0)	40 (40)		
		Non-metastatic	146	42.3 (26.0)	40 (40)	Mann–Whitney *z* = –1.03	0.30
		Metastatic	19	48.4 (26.1)	50 (40)		
Absenteeism by age group		24–43 yrs	25	43.5 (45.7)	20 (100)	KW χ^2^(4) = 3.11	0.54
		44–48 yrs	43	27.7 (42.4)	0 (75)		
		49–54 yrs	42	38.1 (44.8)	12.5 (100)		
		55–59 yrs	23	30.0 (43.9)	0 (100)		
		60–65 yrs	19	45.8 (48.9)	20 (100)		
Presenteeism by age group		24–43 yrs	26	35.8 (31.9)	35 (70)	KW χ^2^(4) = 1.11	0.89
		44–48 yrs	43	33.5 (27.8)	30 (40)		
		49–54 yrs	44	38.6 (33.5)	40 (55)		
		55–59 yrs	24	38.8 (31.7)	40 (60)		
		60–65 yrs	19	41.6 (27.7)	40 (30)		
Overall work productivity loss by age group		24–43 yrs	28	45.7 (41.2)	40 (100)	KW χ^2^(4) = 1.56	0.82
		44–48 yrs	44	41.5 (38.9)	40 (100)		
		49–54 yrs	47	43.3 (40.7)	40 (100)		
		55–59 yrs	25	40.8 (41.5)	40 (100)		
		60–65 yrs	21	47.2 (39.1)	40 (100)		
Activity impairment by age group		24–43 yrs	28	37.5 (24.3)	40 (40)	KW χ^2^(4) = 2.35	0.67
		44–48 yrs	44	42.7 (25.1)	40 (40)		
		49–54 yrs	47	42.8 (28.7)	40 (40)		
		55–59 yrs	25	45.6 (28.6)	40 (40)		
		60–65 yrs	21	48.1 (21.4)	50 (40)		

Note: WPAI:GH scores are percentages ranging from 0 to 100, with higher values indicating greater impairment. Overall work productivity loss combines absenteeism and presenteeism according to standard WPAI scoring. IQR, interquartile range; SD, standard deviation.

**Table 5:** Informal-care receipt and estimated annual informal-care days among breast cancer patients.

Measure	Group	*n*	Mean (SD)/%	Median (IQR)	Test	Statistic	*p*-Value
Informal care receipt (yes%)	Entire sample	236	54.7% received care	—			
	Non-metastatic	192	52.1%	—	Mann–Whitney U	z = –1.66	0.10
	Metastatic	44	65.9%	—			
	24–43 yrs	35	57.1%	—	Kruskal–Wallis	χ^2^(4) = 2.26	0.69
	44–48 yrs	53	56.6%	—			
	49–54 yrs	56	51.8%	—			
	55–59 yrs	41	46.3%	—			
	60–65 yrs	51	60.8%	—			
Estimated annual work-days of informal care received	Entire sample	236	22.5 (33.4) days/year	9 (0–36)			
	Non-metastatic	192	21.3 (32.2)	9 (0–36)	Mann–Whitney U	z = –1.19	0.23
	Metastatic	44	27.8 (37.1)	12 (0–36)			
	24–43 yrs	35	20.6 (31.4)	9 (0–36)	Kruskal–Wallis	χ^2^(4) = 1.43	0.84
	44–48 yrs	53	22.1 (32.7)	9 (0–36)			
	49–54 yrs	56	23.9 (35.8)	12 (0–90)			
	55–59 yrs	41	20.1 (33.6)	9 (0–90)			
	60–65 yrs	51	25.7 (34.1)	12 (0–90)			

Note: Informal care was derived from self-reported care frequency during the previous 12 months and converted to annual work-days assuming 2 h per care episode and an 8 h work-day. Sample sizes vary because of item non-response. IQR, interquartile range; SD, standard deviation.

**Table 6:** Deterministic one-way sensitivity analysis of the extended welfare-inclusive burden estimate.

Parameter Varied	Base-Case Input	Low/High Input Used in OWSA	Low Scenario	High Scenario	Range
WPAI annualisation/productivity persistence	100% of annualised WPAI-derived productivity loss	75% vs. 125% of base-case annualised productivity loss (±25%)	€9616	€13,282	€3666
WTP threshold per QALY	€17,000/QALY	€12,750 vs. €21,250/QALY (±25%)	€10,812	€12,087	€1275
Hourly wage	€8.70/h	€6.53 vs. €10.88/h (±25%)	€9224	€13,674	€4450
Informal-care duration per episode	2 h/episode	1.5 h vs. 2.5 h/episode (±25%)	€11,058	€11,841	€783
Annual working hours	1920 h/year	1440 vs. 2400 h/year (±25%)	€9616	€13,282	€3666
Working-age prevalent population (national burden only)	61% of 5-year prevalent cases (~7500 women)	45.75% vs. 76.25% of 5-year prevalent cases (±25%)	€64.3 million	€107.2 million	€42.9 million

Note: Except for the working-age prevalent population scenario, low and high scenarios are expressed as EUR per patient-year. The working-age prevalent population parameter affects only the illustrative national estimate. For the productivity-persistence row, 125% represents a stress-test multiplier around the base-case annualised loss, not literal persistence beyond 100%.

## Data Availability

The data presented in this study are available on request from the corresponding author due to ethical and privacy restrictions related to human participant data.

## Supplementary Materials

The following supporting information can be downloaded at: https://www.mdpi.com/article/10.3390/healthcare14121693/s1, Table S1. Participant flow and available-case denominators. Table S2. Scenario-based annual monetary burden estimates under base-case assumptions. Table S3. Exploratory subgroup analyses. Table S4. Exploratory multivariable models. Table S5. Completer versus non-completer comparisons. Table S6. STROBE checklist for cross-sectional studies.
