# Supplementary material for: The Societal Burden of Breast Cancer in Working-Age Women in Croatia: A Multicentre Cross-Sectional Study
Source: Healthcare (Basel). 2026 Jun 12;14(12):1693. doi: 10.3390/healthcare14121693 (PMC13299876; doi:10.3390/healthcare14121693)
Supplement: Supplementary file 1 [file healthcare-14-01693-s001.zip › healthcare-4320026-supplementary.pdf]

Table S1. Participant flow and available-case denominators

Flow item	n	Notes
Hospital-record screening	Not systematically recorded	Screened/contacted/refused counts were not available in the database.
Eligible age range for working-age analyses (18–65 years)	271	
EQ-5D-5L / EQ-VAS complete among age-eligible records	233	Complete five EQ-5D-5L dimensions and EQ-VAS.
Currently employed among age-eligible records	165	Includes participants on sick leave.
WPAI absenteeism item available	152	Hours missed due to health problems.
WPAI presenteeism item available	156	Health-related impairment while working.
WPAI absenteeism + presenteeism complete	147	Complete case for standard overall WPAI productivity formula.
WPAI activity impairment available	165	Question on regular daily activities.
Informal-care module available among age-eligible records	233	Receipt and/or frequency of informal care.

Table S2. Scenario-based annual monetary burden estimates under base-case assumptions*

Component	Base-case mean/model-based estimates	Approx. 95% CI	
Monetised HRQoL / welfare loss	€2,550	€2,083 to €3,017	The CI was calculated from the 95% CI for the utility decrement, using 0.150 ± 1.96 × SE, where SE = √(SD_patient²/n_patient + SD_general population²/n_general population). The resulting limits were multiplied by €17,000/QALY.
Productivity loss	€7,333	€6,311 to €8,355	The CI was calculated by applying the lower and upper limits of the productivity-loss estimate to fixed annual working hours and wage values: productivity loss × 1,920 h × €8.70.
Informal-care cost	€1,566	€1,269 to €1,863	The CI was calculated by applying the lower and upper limits of the informal-care days estimate to fixed time and wage values: care days × 8 h × €8.70.
Economic indirect costs	€8,899	€7,835 to €9,963	The CI combines uncertainty around productivity loss and informal-care cost using the square-root-of-sums-of-squares method, assuming independent component uncertainty.
Extended welfare-inclusive societal burden, scenario-based	€11,449	€10,287 to €12,611	The CI combines uncertainty around economic indirect costs and monetised HRQoL loss using the square-root-of-sums-of-squares method, assuming independent component uncertainty.
Illustrative national extended burden	€85.9 million	€77.2 million to €94.6 million	The CI was obtained by multiplying the individual-level lower and upper CI limits by the assumed prevalent population of approximately 7,500 working-age women.

* Under the base-case assumptions, the estimated annual indirect economic cost is about €8,900 per working-age woman with breast cancer. Adding a monetised quality-of-life component increases the estimate to about €11,400, but this broader figure should be treated as illustrative because it may overlap with the economic cost components and depends strongly on valuation assumptions.

Table S3. Exploratory subgroup analyses

Subgroup	Outcome	Mean (SD) by group	p-value
Metastatic status	EQ-5D utility	Non-metastatic: n=190, 0.77 (0.22); Metastatic: n=43, 0.70 (0.27)	0.029
Metastatic status	EQ-VAS	Non-metastatic: n=190, 67.96 (22.52); Metastatic: n=43, 58.23 (24.16)	0.010
Metastatic status	WPAI activity impairment (%)	Non-metastatic: n=146, 42.26 (26.04); Metastatic: n=19, 48.42 (26.09)	0.302
Metastatic status	Annual informal-care cost (€)	Non-metastatic: n=190, €1,466 (€2,299); Metastatic: n=43, €1,984 (€2,538)	0.291
Active/maintenance treatment	EQ-5D utility	Completed/follow-up: n=34, 0.84 (0.14); Active/maintenance: n=199, 0.74 (0.24)	0.034
Active/maintenance treatment	EQ-VAS	Completed/follow-up: n=34, 70.62 (24.51); Active/maintenance: n=199, 65.41 (22.81)	0.171
Active/maintenance treatment	WPAI activity impairment (%)	Completed/follow-up: n=24, 38.33 (25.99); Active/maintenance: n=141, 43.76 (26.06)	0.299
Active/maintenance treatment	Annual informal-care cost (€)	Completed/follow-up: n=34, €1,798 (€2,755); Active/maintenance: n=199, €1,521 (€2,277)	0.574
Time since diagnosis	EQ-5D utility	<2 years: n=75, 0.80 (0.19); 2–5 years: n=91, 0.71 (0.26); >5 years: n=66, 0.78 (0.24)	0.064
Time since diagnosis	EQ-VAS	<2 years: n=75, 67.47 (24.03); 2–5 years: n=91, 64.90 (20.85); >5 years: n=66, 66.53 (25.25)	0.317
Time since diagnosis	WPAI activity impairment (%)	<2 years: n=52, 42.50 (22.91); 2–5 years: n=66, 47.12 (27.83); >5 years: n=46, 37.39 (26.45)	0.158
Time since diagnosis	Annual informal-care cost (€)	<2 years: n=75, €2,167 (€2,618); 2–5 years: n=91, €1,607 (€2,316); >5 years: n=66, €833 (€1,856)	0.000

Table S4. Exploratory multivariable models

Dependent variable	Covariate	Estimate (robust 95% CI)	p-value
EQ-5D utility (n=231)	Age (per 10y)	-0.05 (-0.09 to -0.02)	0.005
	Metastatic	-0.02 (-0.10 to 0.06)	0.615
	Active/maintenance	-0.09 (-0.15 to -0.02)	0.009
	Time since diagnosis	-0.00 (-0.02 to 0.02)	0.934
	Higher education	0.04 (-0.02 to 0.10)	0.158
	Split centre	-0.03 (-0.09 to 0.03)	0.403
EQ-VAS (n=231)	Age (per 10y)	-2.67 (-7.29 to 1.95)	0.258
	Metastatic	-7.09 (-15.89 to 1.71)	0.114
	Active/maintenance	-3.33 (-12.40 to 5.73)	0.471
	Time since diagnosis	0.15 (-0.97 to 1.26)	0.797
	Higher education	0.76 (-5.69 to 7.21)	0.818
	Split centre	-4.69 (-11.05 to 1.67)	0.148
WPAI activity impairment (%) (n=163)	Age (per 10y)	3.86 (-0.93 to 8.66)	0.114
	Metastatic	7.70 (-6.02 to 21.41)	0.271
	Active/maintenance	0.77 (-10.78 to 12.32)	0.896
	Time since diagnosis	-1.79 (-3.51 to -0.08)	0.040
	Higher education	-11.02 (-19.34 to -2.70)	0.009
	Split centre	-1.21 (-9.59 to 7.18)	0.778
Informal care receipt (n=231)	Age (per 10y)	OR 1.00 (0.71 to 1.39)	0.982
	Metastatic	OR 2.33 (0.99 to 5.48)	0.053
	Active/maintenance	OR 1.15 (0.56 to 2.36)	0.709
	Time since diagnosis	OR 0.85 (0.76 to 0.95)	0.005
	Higher education	OR 0.93 (0.54 to 1.60)	0.782
	Split centre	OR 0.98 (0.58 to 1.68)	0.956
Informal care cost (€) (n=231)	Age (per 10y)	263.77 (-114.66 to 642.20)	0.172
	Metastatic	734.00 (-193.48 to 1661.47)	0.121
	Active/maintenance	-535.98 (-1505.24 to 433.28)	0.278
	Time since diagnosis	-176.13 (-271.07 to -81.19)	0.000
	Higher education	-128.06 (-760.17 to 504.05)	0.691
	Split centre	366.36 (-236.63 to 969.35)	0.234

Table S5. Completer versus non-completer comparisons

Module	Variable	Completers	Non-completers	p-value
EQ-5D/VAS module	Age, years	n=233, 51.43 (8.28)	n=33, 50.73 (10.17)	0.988
EQ-5D/VAS module	Time since diagnosis, years	n=232, 3.86 (3.22)	n=33, 4.91 (5.33)	0.724
EQ-5D/VAS module	Metastatic disease	n=233, 43/233 (18.5%)	n=33, 8/33 (24.2%)	0.429
EQ-5D/VAS module	Active/maintenance treatment	n=233, 199/233 (85.4%)	n=33, 21/33 (63.6%)	0.002
EQ-5D/VAS module	Split centre	n=232, 131/232 (56.5%)	n=29, 14/29 (48.3%)	0.403
WPAI absenteeism/presenteeism complete	Age, years	n=147, 49.67 (7.47)	n=18, 50.89 (9.42)	0.261
WPAI absenteeism/presenteeism complete	Time since diagnosis, years	n=146, 3.64 (2.62)	n=18, 3.38 (2.18)	0.858
WPAI absenteeism/presenteeism complete	Metastatic disease	n=147, 15/147 (10.2%)	n=18, 4/18 (22.2%)	0.133
WPAI absenteeism/presenteeism complete	Higher education	n=147, 86/147 (58.5%)	n=18, 5/18 (27.8%)	0.013
WPAI absenteeism/presenteeism complete	Employed	n=147, 147/147 (100.0%)	n=18, 18/18 (100.0%)	1.000
WPAI absenteeism/presenteeism complete	Active/maintenance treatment	n=147, 123/147 (83.7%)	n=18, 18/18 (100.0%)	0.078
WPAI absenteeism/presenteeism complete	Split centre	n=146, 75/146 (51.4%)	n=18, 11/18 (61.1%)	0.435
Informal care module	Age, years	n=233, 51.43 (8.28)	n=33, 50.73 (10.17)	0.988
Informal care module	Time since diagnosis, years	n=232, 3.86 (3.22)	n=33, 4.91 (5.33)	0.724
Informal care module	Metastatic disease	n=233, 43/233 (18.5%)	n=33, 8/33 (24.2%)	0.429
Informal care module	Active/maintenance treatment	n=233, 199/233 (85.4%)	n=33, 21/33 (63.6%)	0.002
Informal care module	Split centre	n=232, 131/232 (56.5%)	n=29, 14/29 (48.3%)	0.403

Table S6. STROBE checklist for cross-sectional studies

STROBE item	Where addressed
Title/abstract	Title and abstract identify the study as a multicentre cross-sectional study.
Background/rationale	The Introduction explains the evidence gap in Croatia/CEE and why non-medical burden is policy relevant.
Objectives	The objective is stated as estimating economic indirect costs and monetised utility/welfare loss.
Study design	The Methods describe the multicentre cross-sectional design and 2024 data collection.
Setting	The two tertiary oncology centres in Split and Zagreb are stated.
Participants	Eligibility, exclusions, recruitment and the lack of screened/declined counts are clarified.
Variables	HRQoL, WPAI, informal care, costs and national extrapolation variables are defined.
Data sources/measurement	The general-population comparator, Slovenian EQ-5D-5L tariff, WPAI:GH translation and informal-care conversion are described.
Bias	Selection bias, tertiary-centre recruitment, missingness and annualisation limitations are discussed.
Study size	The lack of an a priori power calculation is stated, and precision and exploratory subgroup analyses are discussed.
Quantitative variables	Age groups, treatment-phase proxy, time since diagnosis and costing assumptions are described.
Statistical methods	Available-case analysis, non-parametric tests, CIs, OWSA, subgroup analyses and exploratory regressions are described.
Participants/results	Participant flow and available-case denominators provided in Table S1.
Descriptive data	Table 1 is retained, and missing denominators are explained.
Outcome data	Main outcomes are retained, with uncertainty intervals added.
Main results	Economic indirect costs and HRQoL/welfare losses reported separately.
Other analyses	Sensitivity analysis is provided in main-text Table 6; subgroup, multivariable and completer/non-completer analyses are provided in Supplementary Tables S3–S5.
Discussion/key results	The main findings are framed as scenario-based estimates.
Limitations	Double counting, annualisation, WPAI:GH attribution, informal care assumptions, and selection/missingness limitations expanded.
Interpretation	The policy interpretation is cautious and linked to methodological uncertainty.
Generalisability	Tertiary-centre recruitment and transferability to Croatia/CEE are discussed.
Funding	The funding statement is retained.
